# Supplementary material for: Effect of Hypoxia on Pulmonary Endothelial Cells from Bleomycin-Induced Pulmonary Fibrosis Model Mice
Source: Int J Mol Sci. 2022 Aug 12;23(16):8996. doi: 10.3390/ijms23168996 (PMC9408900; doi:10.3390/ijms23168996)
Supplement: Supplementary file 1 [file ijms-23-08996-s001.zip › ijms-1808926-Supplementary.pdf]

Additional files

Additional file 1: Figure S1. Pathological image of intratracheal bleomycin-induced pulmonary fibrosis

Representative optical microscopy images of mouse lungs. Hematoxylin-eosin stain for saline (A) and bleomycin-treated lungs at day 7 (B). Scale bars indicate 500 μm (20 μm in inset). Masson's trichrome stained saline (C) and bleomycin-treated lungs at day 7 (D). Scale bars indicate 20 μm.

Additional file 2: Table S1. Primers used for quantitative real-time PCR

	Forward	Reverse
vWF	TTGGGAACTCCTGGAAAGTG	GATGTTGTTGTGGCAAGTGG
MMP-12	TGATGCAGCTGTCTTTGACC	TGGGAAGTGTGTGGAAATCA
PAI-1	AGGATCGAGGTAAACGAGAGC	GCGGGCTGAGATGACAAA
TGF-β1	TGGAGCAACATGTGGAACTC	CAGCAGCCGGTTACCAAG
CTGF	GAGTGTGCACTGCCAAAGAT	GGCAAGTGCATTGGTATTTG
PDGF-A	GGAACTGAACAGGTGGGAGA	ATTCCACGTAAGGCCATCAG
PDGF-B	ATGTGCCCTTCAGTCTGCTC	GAGACAGGTCTCCTGCCCTA
PDGF-C	GCCCGAAGTTTCCTCATACA	ACACTTCCATCACTGGGCTC

PDGF-D	CGAGGGACTGTGCAGTAGAAA	TTGATGGATGCTCTCTGCGG
eNOS	TCCGGAAGGCGTTTGATC	GCCAAATGTGCTGGTCACC
iNOS	CACCTTGGAGTTCACCCAGT	ACCACTCGTACTTGGGATGC
α-SMA	TGTGCTGGACTCTGGAGATG	GAAGGAATAGCCACGCTCAG
Twist-1	CGCACGCAGTCGCTGAACG	GACGCGGACATGGACCAGG
Snail	CCACTGCAACCGTGCTTTT	GTGCTTGTGGAGCAAGGAC
Slug	TACAGCCCCATCACTGTGTGGAC	CGCCCCAAAGATGAGGAGTATCC
β - actin	AAGGCCAACCGTGAAAAGAT	GTGGTACGACCAGAGGCATAC

vWF, von Willebrand factor; MMP-12, matrix metalloproteinase 12; PAI-1, plasminogen activator inhibitor 1; TGF-β, transforming growth factor-β; CTGF, connective tissue growth factor; PDGF, platelet-derived growth factor; iNOS, inducible nitric oxide synthase; eNOS, endothelial nitric oxide synthase; α-SMA, α-smooth muscle actin. Sequences are shown as (left to right) 5' to 3'.
