# Supplementary figures and images for: Effect of Hypoxia on Pulmonary Endothelial Cells from Bleomycin-Induced Pulmonary Fibrosis Model Mice
Source: Int J Mol Sci. 2022 Aug 12;23(16):8996. doi: 10.3390/ijms23168996 (PMC9408900; doi:10.3390/ijms23168996)

Figure S1. Pathological image of intratracheal bleomycin-induced pulmonary fibrosis

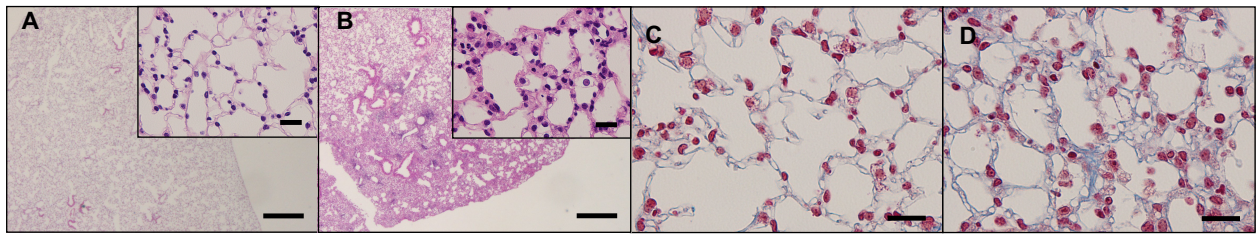

Supplement: Supplementary file 1 [file ijms-23-08996-s001.zip › ijms-1808926-Figure S1.pdf]
